# Bio-Inspired PG/PEI Co-Deposition for Interfacial Modification of HMX/F2602

**DOI:** 10.3390/polym17121702

**Published:** 2025-06-19

**Authors:** Ningxin Ma, Wenzheng Xu, Xiaolong Chang, Shuying Lan

**Affiliations:** School of Environment and Safety Engineering, North University of China, Taiyuan 030051, China; 18135194320@163.com (N.M.); cxl15513000215@163.com (X.C.); l1902131652@163.com (S.L.)

**Keywords:** HMX, crosslinking polymerization, mechanical sensitivity

## Abstract

The issue of interfacial inhomogeneity in energetic materials remains a significant challenge. In this study, fluoroelastomer F2602 was applied to HMX crystals using a water suspension granulation technique, followed by a bio-inspired coating formed via the crosslinking polymerization of polyethyleneimine (PEI) and pyrogallol (PG) on the HMX/F2602 composite. This process resulted in the formation of an HMX/F2602/PEI-PG microcapsule structure. Various characterization techniques confirmed that the chemical structure and polycrystalline morphology of the crystals were preserved throughout the coating process, maintaining the characteristic β-HMX morphology. The introduction of the PG–PEI shell significantly improved the coating coverage and minimized the exposure of crystal surfaces. Furthermore, compared to HMX/F2602, the HMX/F2602/PEI-PG composite exhibited notably enhanced thermal stability and reduced mechanical sensitivity. These improvements are attributed to the advantageous effects of the microcapsule structure formed by the bio-inspired coating on the material’s properties.

## 1. Introduction

Due to the high sensitivity of energetic materials, their application in production environments is greatly restricted, making the enhancement of their safety a top priority. As a type of energetic material, HMX has been widely used in military explosives due to its high detonation velocity, detonation heat, and powerful explosive performance [1,2,3]. However, factors such as production processes and environmental conditions contribute to structural defects in HMX, increasing its sensitivity. To improve its safety and practicality, various modification methods have been explored. Among them, layer-by-layer assembly [4] and surface grafting techniques [5,6,7] are relatively complex and have low success rates. Therefore, this study adopts a simpler and more efficient approach: applying a dense coating to the surface of HMX through in situ polymerization. This method effectively reduces its sensitivity, making the explosive less susceptible to external stimuli and accidental detonation.

In recent years, bio-inspired coatings have attracted increasing attention due to their potential to enhance the chemical stability and impact resistance of various substrates. Messersmith et al. demonstrated that dopamine can oxidize under alkaline conditions, forming a strong, polymer-like coating on a wide range of substrates [8,9,10]. In situ polymerization methods can effectively enhance the thermal stability of energetic materials, reduce their sensitivity to external stimuli, and improve mechanical strength. The polymer layer can effectively isolate energetic compounds from environmental factors, control energy release behavior, and significantly enhance the safety and service life of such materials. Li et al. self-polymerized dopamine on the surface of CL-20 to prepare a core–shell structure, which greatly reduced its mechanical sensitivity and enhanced thermal stability [11]. Yang et al. formed a coating on the surface of HMX by in situ polymerization, which greatly reduced its friction sensitivity and improved its thermal stability and mechanical properties [12]. Zheng et al. coated polyphenol tannic acid (TA) on the surface of HMX by in situ polymerization, which effectively reduced its impact and friction sensitivity and improved its mechanical behavior. However, tannic acid (TA) and polydopamine (PDA) are expensive and environmentally friendly [13,14,15]. Bioadhesives inspired by marine invertebrates have attracted much attention [16,17]. Bioadhesives contain abundant pyrogallol groups, which are crucial to their strong adhesion properties. Polymers containing pyrogallol and pyrogallol contribute greatly to the adhesion force, so a substrate-independent surface coating can be achieved (the adhesion force of the polymer containing pyrogallol is about seven times higher than that of the polymer containing pyrogallol) [18,19,20]. Polyphenol pyrogallols and derivative quinones can covalently crosslink-NH2 groups in alkaline solutions to simulate strong bioadhesion [21,22,23]. The structure of pyrogallol (PG) is similar to that of DA and TA. It is composed of three-OH groups in a molecule, namely gallic acid groups, which makes it easier to self-polymerize to form adhesion coatings, and is more stable than TA due to its ester-free structure. The introduction of polyethyleneimine (PEI) can promote the uniform polymerization and deposition of PG, so as to reduce the cluster phenomenon of PG self-polymerization and make the whole coating more compact and stable. Therefore, the combination of PG and amine-containing PEI to prepare an effective coating is considered to be an effective modification strategy.

Surface coating represents one of the most straightforward and efficient approaches to lowering HMX sensitivity. Thanks to the continuous efforts of researchers, numerous coating techniques have been developed, including the water suspension method, high-temperature evaporation method, vapor precipitation method, spray drying method, and in situ polymerization method [24,25,26,27,28,29,30]. Compared with traditional granulation methods, the water suspension granulation method exhibits significant advantages. Primarily, by using water as the dispersing medium, this approach significantly reduces the risk of electrostatic discharge and frictional heating during processing, thereby enhancing operational safety and minimizing the likelihood of accidental ignition or explosion. Furthermore, the thermal buffering effect of water helps maintain a controlled temperature environment, preventing thermal decomposition or degradation of heat-sensitive energetic components. This method also enables the formation of granules with uniform particle size distribution and dense microstructures, which contribute to improved flowability, packing density, and consistent energy release performance. In addition, the suppression of dust generation further enhances safety during handling, making this technique highly adaptable for the large-scale and safe manufacturing of energetic materials.

In this study, inspired by the strong adhesive capabilities of mussels and tunicates, PG and PEI were combined into a synergistic biomimetic coating system and applied to energetic materials via in situ polymerization. Experimental results show that the PEI-PG coating forms a uniform layer on the substrate, improving surface uniformity, enhancing thermal stability, and reducing mechanical sensitivity. Characterization analysis confirms that this PEI-PG coating strategy is simple, efficient, and effective for interfacial modification of energetic materials.

## 2. Experiment

### 2.1. Materials

HMX was supplied by Gansu Yinguang Chemical Group Co., Ltd. (Baiyin, China). Laboratory-synthesized HMX with a particle size of 1–10 μm was used throughout all experimental procedures. Tris(hydroxymethyl)aminomethane was obtained from Guangzhou Jiangshun Chemical Technology Co., Ltd. (Guangzhou, China). Pyrogallol (PG) and polyethyleneimine (PEI) were purchased from Shanghai McLean Biochemical Technology Co., Ltd. (Shanghai, China). Sodium periodate and fluoroelastomer F2602 were provided by Yuyao Fluorine New Materials Co., Ltd. (Jiangxi, China). Ethyl acetate was acquired from Tianjin Fuyu Fine Chemical Co., Ltd. (Tianjin, China)., concentrated hydrochloric acid from Aladdin Reagent Co., Ltd. (Shanghai, China)., and deionized water from Taiyuan Lanmiao Water Industry (Taiyuan, China).

### 2.2. Sample Preparation

#### 2.2.1. Preparation of HMX/F2602

**Figure 1:**
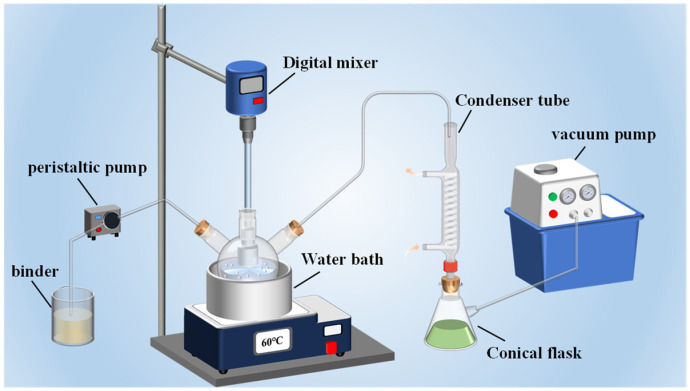
Device diagram of water suspension coating technology.

The preparation steps of HMX/F2602 are shown in Figure 1. Firstly, 4.75 g of HMX was weighed and mixed with 50 mL of deionized water and ultrasonically stirred for 25 min at room temperature to form a homogeneously dispersed white suspension. The mixture was then heated to 50 °C in a water bath. Next, 5.25 g of a 5 wt.% F2602/CH3COOCH2CH3 binder solution was added dropwise while adjusting the stirring speed to facilitate particle formation and stabilization. As the temperature reached 60 °C, vacuum filtration was performed until the particles were morphologically intact. The particles formed HMX/F2602 (F2602, 5 wt.%) after filtration, washing, and drying.

#### 2.2.2. Preparation of HMX/F2602/PG-PEI

**Figure 2:**
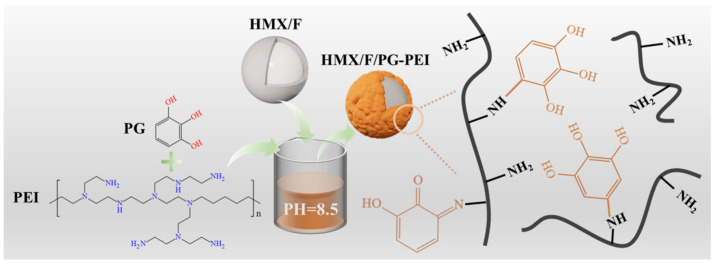
Mechanism of HMX/F2602/PG-PEI preparation.

HMX/F2602/PG-PEI was prepared as shown in Figure 2. Firstly, 1.2114 g of Tris (hydroxymethyl)aminomethane was dissolved in 100 mL of deionized water to prepare a 0.1 mol/L Tris buffer solution. Hydrochloric acid was added dropwise to adjust the pH to 8.5, forming a Tris-HCl buffer. Subsequently, 0.12 g of catechol was introduced into the solution under ultrasound-assisted stirring, followed by the gradual addition of 0.12 g of polyethyleneimine solution. Finally, 4.75 g of HMX/F2602 composite particles and 0.3 g of sodium periodate were incorporated, and the mixture was stirred at room temperature for 8, 16, and 24 h. After filtration, washing, and drying, energy-containing composite particles in the form of microcapsules (HMX/F2602PG-PEI) were obtained.

### 2.3. Characterization

The morphology of the samples was analyzed using scanning electron microscopy (SEM) (Zeiss Sigma 300, Carl Zeiss AG, Oberkochen, Germany). The crystal morphology and structure were analyzed by an X-ray diffractometer (X ‘Pert PRO MPD, PANalytical B.V., Almelo, The Netherlands). The elemental composition, chemical bonding, and coating coverage of the samples were assessed by XPS (Axis Ultra DLD, Kratos Analytical Ltd., Manchester, UK). The thermal stability of the samples was determined by a DSC 3500 Sirius differential scanning calorimeter (NETZSCH-Gerätebau GmbH, Selb, Germany).

### 2.4. Sensitivity Test

In this study, the BAM impact sensitivity tester (Model BFH-12, Addison) was employed following the standard procedure outlined in GB/T 21567 [31], “Impact Sensitivity Test Method for Dangerous Goods and Explosives.” The experimental parameters were set as follows: impact energy (E, J) was calculated using the formula E ≈ M × g × H; the mass of each test sample was 40 mg; the relative humidity was maintained at 60%; the laboratory temperature was controlled at 25 °C; and the samples were dry molded powders. To ensure data reliability, each test was repeated five times.

Similarly, with reference to GB/T21566 [32] “Test method for friction sensitivity of dangerous goods and explosives,” this study used the BAM type friction sensitivity test device (model FSKM10, Addisain company production, Beijing, China). The experimental conditions were set as follows: the friction load (N) was applied; and the mass of each experimental sample was 10 mg. The relative humidity of the environment was maintained at 60%; the laboratory temperature was 25 °C; the samples used were dry molding powder; and in order to obtain stable and repeatable data, the friction sensitivity test was repeated six times.

### 2.5. Static Compression Test

The tests were conducted using a universal testing machine (Instron Model TM-30, Norwood, MA, USA), following the GB1041-1992 standard [33]. The test conditions were set at a temperature of 22.00 °C, a humidity of 14.00%, and a test speed of 0.5 mm/min. The sample, with dimensions of φ 9.5 mm × 10 mm and a density of 1.7 g/cm^3^, was subjected to testing. The pressure–time curve was recorded using the compression testing machine, and the stress–strain curve was derived through coordinate transformation.

## 3. Results and Discussion

### 3.1. Morphology

**Figure 3:**
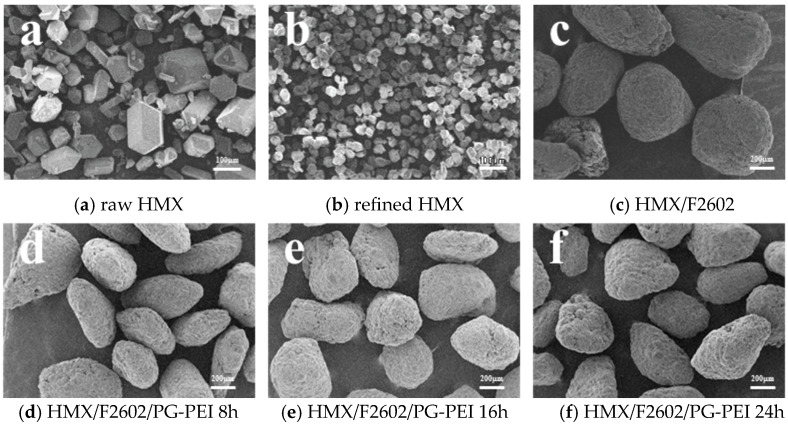
SEM image of the sample.

Figure 3 below shows the SEM images of the samples. Among them, Figure 3a exhibits the raw material HMX, which has a smooth surface and shows a typical β-shaped polyhedral prism structure, indicating good purity and crystallinity. However, it is evident that the particle size is not uniform, and the distribution of particle sizes is broad. Figure 3b shows the raw material HMX after refinement, which clearly exhibits a uniform and spherical particle size, uniform particle size distribution, and no obvious agglomeration effect. Figure 3c shows the HMX after fluoropolymer binder coating, from which it can be seen that its surface is rough and uneven, with a strong sense of granularity and an overall lyotropic morphology. This is due to the fact that the binder solution completely wets the energy-containing particles when the preparation is carried out, and when the solvent evaporates, the binder adheres to the surface of the explosive particles to form small PBX particles, and these small PBX particles bond with each other to form large PBX particles. It is not difficult to see that there are some microcracks and pores on the surface, which are caused by the uneven distribution of the binder during the preparation process and the friction collision of the HMX particles with the stirring paddle or with the inner wall of the beaker during the stirring process. Figure 3d–f are the snapshots of sample coating electron microscopy at 8 h, 16 h and 24 h, respectively. It can be seen that the PG-PEI composite coating was uniformly coated onto the HMX-F particles, and with the increase in the coating time, its coating shell gradually became uniform and dense, and the cracks and small pores appeared on the surface became less prominent. This indicates that the PG-PEI coating effectively fills the defects on the surface and has a positive effect on improving the morphological characteristics of PBX.

### 3.2. Chemical Properties

**Figure 4:**
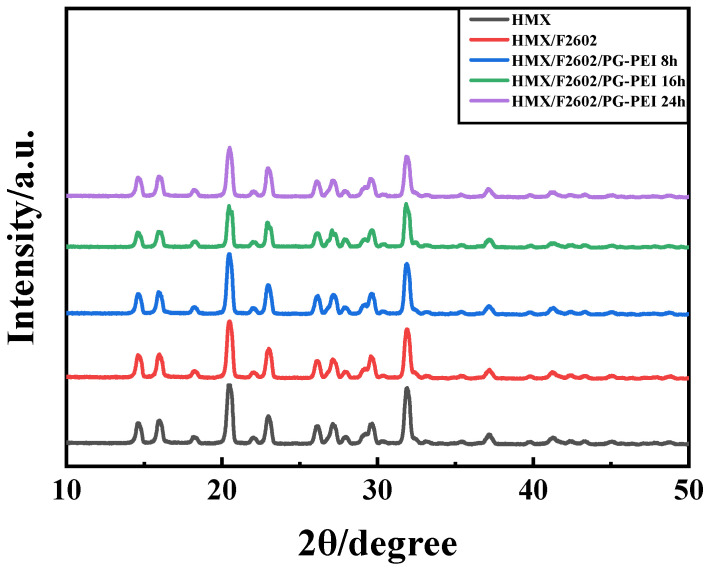
XRD patterns of samples.

Among the four crystal forms of HMX, β-HMX has the best physical and chemical properties, detonation performance, stability, and mechanical sensitivity, making it the safest option [34,35]. Figure 4 shows the XRD results of HMX crystals, HMX/F2602, and the corresponding coated particles. The diffraction peaks of HMX crystals match those of the standard β-HMX X-ray diffraction pattern (JCPDS, No. 45-1539). Peaks at 16.0°, 20.4°, 23.0°, 29.6°, 31.9°, and 41.2° correspond to the crystal planes (-111), (-102), (120), (022), (1-32), and (023), respectively. The diffraction patterns of HMX/F2602 and HMX/F/PG-PEI only show minor shifts compared to HMX, with no new peaks, indicating that the phase of HMX remains unchanged during the coating process using water suspension and in situ polymerization methods, and the crystalline phase is stable throughout.

### 3.3. Surface Analysis

**Figure 5:**
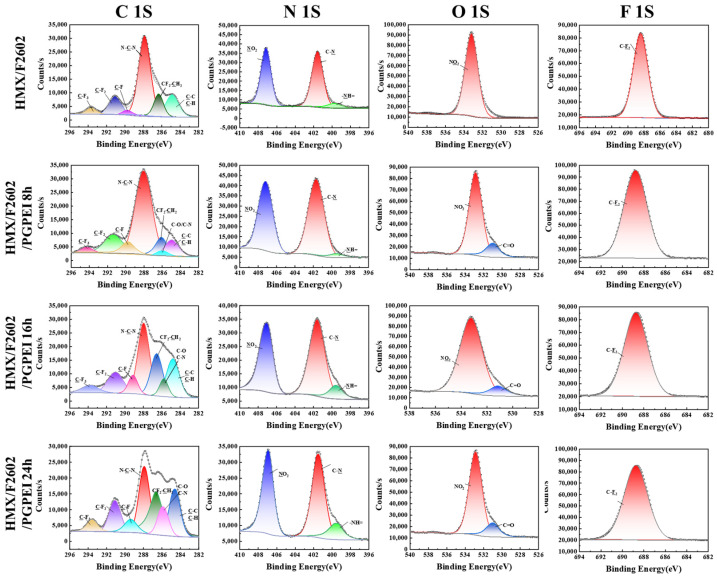
High-resolution XPS spectra of C 1s, N 1s, O 1s, and F 1s regions of samples.

To further investigate the structural properties of the energy-containing composite particles, X-ray photoelectron spectroscopy (XPS) was used to analyze the elemental composition and chemical states of elements in the 10 nm surface region of several samples. High-resolution spectra were processed using Advantage XPS software (version 5.948) to obtain the XPS profiles for C 1s, N 1s, O 1s, and F 1s, as illustrated in Figure 5. As reported in the literature [36,37,38], the C 1s spectrum of HMX was fitted with two characteristic peaks: one for the C-C bond at 284.6 eV and another for the N-C-N bond at 287.4 eV. The N 1s spectrum of HMX displayed two peaks, corresponding to the -NO2 bond at 406.4 eV and the C-N bond at 400.9 eV. The O 1s spectrum of HMX exhibited a peak at 532.5 eV associated with -NO2. In contrast, the XPS spectrum of HMX modified with F2602 revealed notable changes in the peak positions and the appearance of new characteristic peaks. These included C-F3, C-F2, C-F, and CF2-CH2 bonds in the C 1s region, and a C-F2 peak in the F 1s region, confirming successful coating of F2602 on the HMX surface. The XPS spectrum of HMX/F/PG-PEI showed similarities to HMX/F2602, but a new peak corresponding to C-O/C-N bonds at 285.73 eV was observed, indicating the involvement of a Michael addition reaction. The N 1s spectrum also revealed the presence of an -NH= bond, while the O 1s spectrum exhibited a new C=O peak. These changes can be attributed to the abundant hydroxyl groups from PG and the amine groups from PEI. The intensity of these new peaks increased with longer coating durations, providing strong evidence for the formation of a shell layer on the PBX surface, which results from the effective noncovalent interactions between the o-triphenol PG and the PEI polymer rich in amines.

To further examine the surface characteristics of the samples, the contents of C 1s, N 1s, O 1s, and F 1s for HMX, HMX/F, and HMX/F/PG-PEI were analyzed and are presented in Table 1. Compared to the unmodified HMX, both HMX/F and HMX/F/PG-PEI showed a significant reduction in intensity. Specifically, the N/C ratio for HMX/F2602 decreased from 1.46 for pure HMX to 0.78, confirming successful coating of F2602 on the HMX crystal surface. When further coated with PG-PEI, the N/C ratio for HMX/F2602/PG-PEI (24 h) dropped to 0.65. These findings suggest that both water suspension and in situ polymer encapsulation methods effectively reduce the exposure of HMX crystals. The additional PG-PEI coating further enhances this encapsulation, potentially improving the safety of the explosives.

### 3.4. Thermal Performance

**Figure 6:**
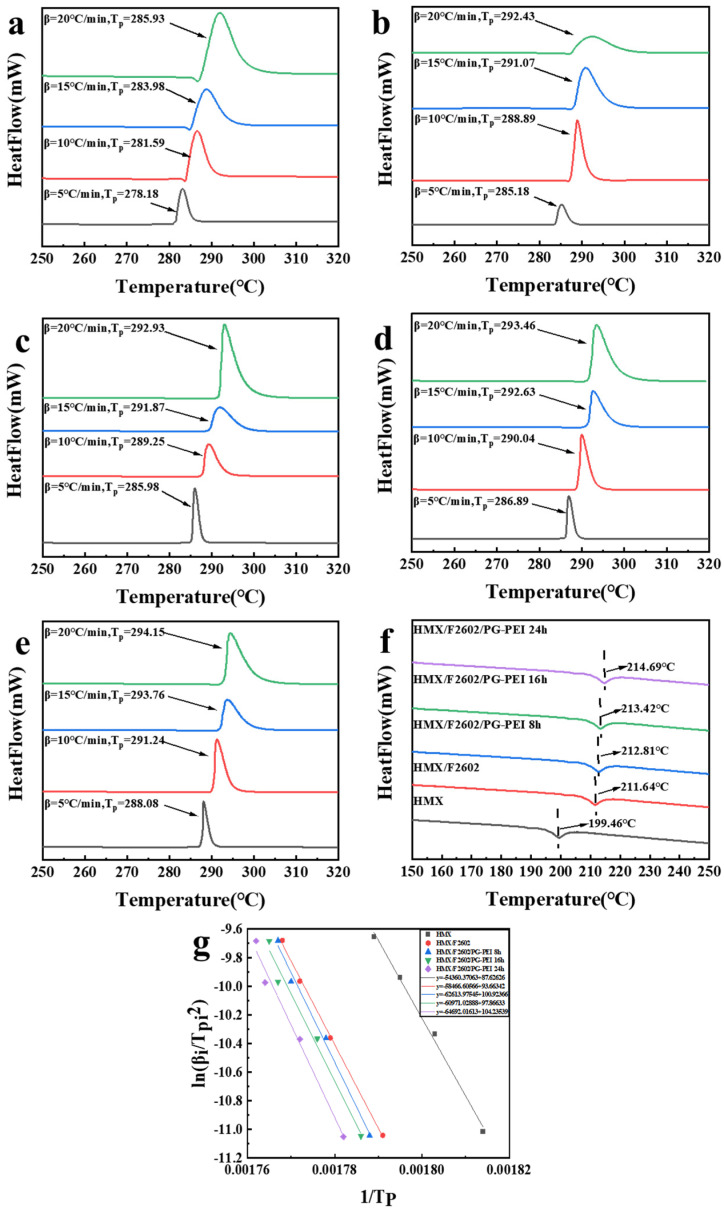
DSC curves of (**a**) HMX, (**b**) HMX/F2602, (**c**) HMX/F2602/PG-PEI 8 h, (**d**) HMX/F2602/PG-PEI 16 h, and (**e**) HMX/F2602/PG-PEI 24 h; (**f**) temperature curve of crystal phase transition at 10  °C heating rates; (**g**) Kissinger fitting curve.

Figure 6 illustrates the changes in original HMX and surface-treated HMX under various heating rates. Compared to HMX, the thermal decomposition peak of HMX/F2602 shifts to higher temperatures, indicating improved thermal resistance. HMX/F2602/PG-PEI shows even an greater delay in thermal decomposition than HMX/F2602, suggesting that both F2602 and PG-PEI coatings can effectively retard the thermal decomposition of HMX, with HMX/F2602/PG-PEI exhibiting superior retardation effects.

The phase transition temperature curves for each sample at a heating rate of 10 °C are displayed in Figure 6d. After encapsulation with F2602, the polycrystalline phase transition temperature slightly increases to 211.64 °C. Further coating with PG-PEI shifts the phase transition temperature to an even higher value, reaching 214.69 °C for HMX/F2602/PG-PEI after 24 h. This increase may be attributed to the dense PG-PEI coating, which can absorb some heat during the early stages of heating, thereby elevating the polycrystalline phase transition temperature of the HMX crystals. Thus, both F2602 and PG-PEI inhibit the phase transition of HMX, with PG-PEI showing a more pronounced effect. This indicates that the coatings play a positive role in enhancing the thermal stability of the explosives.

The Kissinger-Akahira-Sunose (KAS) method is widely employed to determine the kinetic parameters of samples [39], and its calculation formula is as follows: (1) and (2). Additionally, the Zhang-Hu-Sheli Equations (3) and (4) are significant for calculating the thermodynamic parameters of samples [40].

(1) $$\ln\left(\frac{\beta_{i}}{T_{pi}^{2}}\right)=\ln\left(\frac{AR}{E_{a}}\right)-\frac{E_{a}}{RT_{pi}}$$

(2) $$A=\frac{E_{a}\beta }{RT_{P}^{2}}\exp\left(\frac{E_{a}}{RT_{P}}\right)$$

(3) $$T_{p_{i}}=T_{p0}+b\beta_{i}+{c\beta_{i}}^{2}$$

(4) $$T_{b}=\frac{E_{a}-\sqrt{E_{a}^{2}-4RE_{a}T_{p0}}}{2R}$$

In the given equation, $\beta_{i}$ represents the heating rate in °C·min^−1^, $T_{pi}$ denotes the peak decomposition temperature at $\beta_{i}$, A is the pre-exponential factor in $\min^{-1}$, $T_{b}$ is the critical temperature for thermal explosion in °C, $T_{p0}$ is the initial thermal decomposition temperature in °C, R is the gas constant (8.314 J·min^−1^·K^−1^), $E_{a}$ is the apparent activation energy in kJ·min^−1^, and b and c are constants. Using the peak thermal decomposition temperatures for each sample at various heating rates, a linear regression analysis of $1/T_{pi}$ against $ln\left(\beta_{i}/T_{pi}^{2}\right)$ was performed. The results, showing a linear correlation coefficient near 1, underscore the high accuracy of the regression. Employing these linear regression outcomes, the kinetic parameters and reaction kinetics of the thermal decomposition for each sample were determined using the Kissinger method and the Zhanghu sheli equation, as detailed in Table 2.

Compared to HMX, the activation energy $E_{a}$ for HMX/F2602 and HMX/F2602/PG-PEI increased by 36.15 kJ·mol^−1^ and 81.03 kJ·mol^−1^, respectively. This indicates that HMX/F2602/PG-PEI exhibits superior thermal stability. Additionally, the logarithm of the pre-exponential factor log A  for HMX/F2602/PG-PEI is higher than that for both HMX and HMX/F2602, suggesting that the microencapsulated composite material has a weaker thermal decomposition behavior. This further confirms that the coating significantly enhances the thermal stability of the explosive.

### 3.5. Sensitivity Test

**Figure 7:**
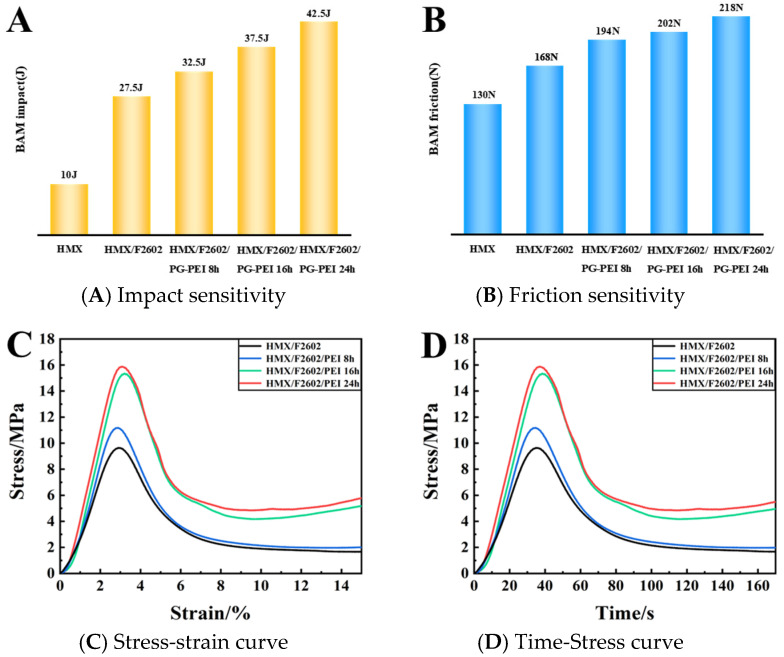
Impact (**A**) and friction sensitivity (**B**) of each sample. (**C**) Stress-strain curve. (**D**) Time-Stress curve.

As indicated in Table 3 and Figure 7A,B, the impact initiation energy increased significantly for both HMX/F2602 and HMX/F2602/PG-PEI compared to pure HMX, with increments of 17.5 J and 32.5 J, respectively. Additionally, the friction threshold rose to 218 N, indicating a notable reduction in mechanical sensitivity. This improvement is primarily attributed to the viscoelastic properties of F2602, which can effectively absorb impact energy and provide a buffering effect. Furthermore, the PG-PEI coating smooths the surface of HMX/F2602, substantially reducing micro-cracks and surface defects, thereby lowering the probability of hotspot formation upon impact. The coating formed via non-covalent cross-linking between PG and PEI also exhibits strong adhesion, which helps to prevent the dislocation or sliding of PBX particles under compression and shear stress, further enhancing the energy dissipation capability of the system.

### 3.6. Static Compressive Strength

The compressive stress-strain and pressure-time images of the HMX/F2602 and HMX/F2602/PG-PEI energetic composites are shown in Figure 7C,D. The maximum pressure of HMX/F2602 is 9.34 MPa. The maximum pressure that PG-PEI can withstand after surface modification is 15.89 MPa, and the static compressive strength is increased by nearly 70%. This is due to the full non-covalent bond assembly between PG-PEI and HMX/F2602, including electro-transfer, hydrogen bonding, etc. The PG-PEI composite network structure formed by gradual polymerization and crosslinking is coated or deposited on the surface of HMX/F2602. The HMX crystal is bonded to the fluorine-containing polymer and PG/PEI forms multiple interface interactions, thereby increasing the yield strength and changing the mechanical behavior.

## 4. Discussion

This study introduces a novel bio-inspired PG-PEI coating technology for modifying the surface interface of HMX/F2602 energetic materials, thereby significantly enhancing their thermal stability and mechanical properties. HMX/F2602 composite particles were prepared via the water suspension method and further coated with a PEI-PG copolymer to transform the surface of HMX crystals into a microencapsulated structure with uniform coating. XRD and SEM characterization results indicate that the β-crystalline form of HMX remained unaltered during the coating process, while the coating exhibited uniform and dense coverage, successfully reducing the surface exposure of HMX crystals.

Thermal analysis demonstrates that the PG-PEI coating significantly improves the thermal stability of HMX, evidenced by a higher initial thermal decomposition temperature and increased activation energy. Compared with pure HMX, the coated samples exhibit stronger inhibition of pyrolysis. Additionally, mechanical sensitivity tests reveal that the impact and friction sensitivities of the coated composites are significantly reduced, attributed to the buffering effect of the PG-PEI coating and enhanced interfacial adhesion.

Static compression tests further confirm that the PG-PEI coating enhances the mechanical strength of HMX/F2602 composites, with a nearly 70% improvement in compressive performance. This enhancement is primarily attributed to multiple interfacial interactions between the PG-PEI coating and HMX/F2602, which strengthen the structural integrity and deformation resistance of the material.

In summary, the bio-inspired coating technology developed in this study provides an effective approach for modifying energetic materials, simultaneously improving thermal stability and reducing mechanical sensitivity, with promising application prospects. Future research will further investigate the long-term environmental stability of the coating and its potential for broader applications.

## Figures and Tables

**Table 1:** The surface elemental concentrations of samples.

Scheme 1.	C 1s (%)	N 1s (%)	O 1s (%)	F 1s (%)	N/C
HMX	26.76	39.11	34.13	-	1.46
HMX/F2602	38.67	30.01	21.43	9.89	0.78
HMX/F2602/PG-PEI 8 h	41.67	29.36	23.41	5.56	0.71
HMX/F2602/PG-PEI 16 h	43.08	28.93	23.68	4.31	0.67
HMX/F2602/PG-PEI 24 h	44.16	29.08	22.09	4.67	0.65

**Table 2:** Kinetic and thermodynamic parameters of samples.

Samples	KAS Method E_a_/kJ·mol^−1^	Log (A/s^−1^)	R^2^	$T_{p0}$/°C	$T_{b}/{}^{\circ}C$
HMX	437.83	42.71	0.99	273.17	278.84
HMX/F2602	473.98	45.73	0.99	279.23	284.57
HMX/F2602/PG-PEI 8 h	484.06	46.67	0.99	282.97	288.27
HMX/F2602/PG-PEI 16 h	507.25	48.88	0.99	284.38	289.45
HMX/F2602/PG-PEI 24 h	518.86	49.92	0.99	285.41	290.39

**Table 3:** Impact and friction sensitivities of samples.

Samples	Content/%	BAM Impact/J	BAM Friction/N
HMX	-	10	130
HMX/F2602	95:5	27.5	168
HMX/F2602/PG-PEI 8 h	95:3:2	32.5	194
HMX/F2602/PG-PEI 16 h	95:3:2	37.5	202
HMX/F2602/PG-PEI 24 h	95:3:2	42.5	218

## Data Availability

All data supporting the findings of this study are included in the article.
